# Measurement and regulation of cardiac ventricular repolarization: from the QT interval to repolarization morphology

**DOI:** 10.1098/rsta.2008.0284

**Published:** 2009-02-27

**Authors:** Jean-Philippe Couderc

**Affiliations:** Heart Research Follow-Up Program, Cardiology Department, Box 653, University of Rochester Medical Center601 Elmwood Avenue, University of Rochester, Rochester, NY 14642, USA

**Keywords:** QT interval, T-wave morphology, vectocardiography, repolarization reserve, electrocardiogram, QT hysteresis

## Abstract

Ventricular repolarization (VR) is a crucial step in cardiac electrical activity because it corresponds to a recovery period setting the stage for the next heart contraction. Small perturbations of the VR process can predispose an individual to lethal arrhythmias. In this review, I aim to provide an overview of the methods developed to analyse static and dynamic aspects of the VR process when recorded from a surface electrocardiogram (ECG). The first section describes the list of physiological and clinical factors that can affect the VR. Technical aspects important to consider when digitally processing ECGs are provided as well. Special attention is given to the analysis of the effect of heart rate on the VR and its regulation by the autonomic nervous system. The final section provides the rationale for extending the analysis of the VR from its duration to its morphology. Several modelling techniques and measurement methods will be presented and their role within the arena of cardiac safety will be discussed.

## 1. Introduction

The genesis and regulation of the electrical activity of the heart and the ability of this organ to provide discontinued demand makes cardiac physiology a fascinating topic. It is the electrical properties of the myocardial cells, their distribution within the heart chambers and their activation time across the geometry of the ventricles, as well as their regulation by the central nervous system, that guarantee the rapid and organized energy transfer required to ensure the normal mental and physical activities of an individual.

In the most recent years, advances in molecular biology and genetics have provided tremendous insights into the functioning of the heart and its regulation. As an example, structural changes in the proteins that control ion fluxes across the plasma membranes of cardiac cells have been linked to profound electrical dysfunctions with pro-arrhythmic effects, such as the short and long QT syndromes (LQTS) or the Brugada syndrome. It is noteworthy that such electrical dysfunctions can be secondary to extrinsic cardiomyopathies, such as in ischaemia, heart failure (with the downregulation of potassium current; Nabauer & Kaab 1998) or following drug therapies. In this last example, drug molecules can bind to receptors inside the ion channel and modify their functioning.

The majority of methods analysing ventricular repolarization (VR) and its regulation have been primarily directed towards the QT interval: its duration, variability and dynamicity. The dynamic and temporal aspects of the QT interval have been elegantly reviewed by Pueyo *et al*. (2009) in a recent issue of this journal. In this review, I will strive to complement Pueyo's review on the QT interval by extending this description to the most recent efforts around the understanding of VR regulation and the analysis of its morphology. I will examine the various factors regulating the VR, and identify the rationale for extending the repolarization analysis from the QT interval to its morphology.

## 2. Physiological factors modulating and regulating cardiac ventricular repolarization

**Figure 1:**
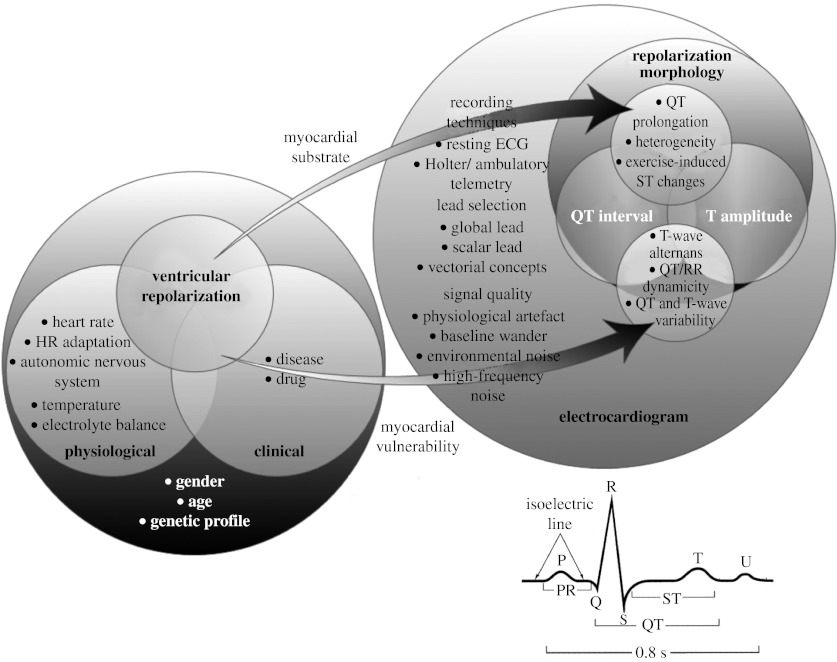
Schema summarizing the list of factors influencing the VR process of the heart. The right part extends the description to the technical factors to be considered when measuring the VR to evaluate the presence of myocardial arrhythmogenic substrate and/or myocardial vulnerability. In this schema, circles with common areas represent an interaction between associated groups of factors. For instance, a drug is defined as a clinical factor that can influence the VR directly or by modifying heart rate (HR), HR adaptation and the autonomic nervous system. In addition, the effect of a drug might be dependent on an individual's age, gender and genetic profile, and his/her electrolyte balance. A typical cardiac beat and its corresponding waves are described in the lower right corner.

The heterogeneity of the morphologies and the activation sequences of the action potentials (AP) generated by cardiac cells between, across and inside the heart ventricles during the repolarization process produce the voltage gradient responsible for the inscription of the T-wave on the surface electrocardiogram (ECG). Measuring cardiac repolarization including QT interval duration, T-amplitude and overall morphology is a challenging task requiring careful consideration of technical, clinical and physiological factors. Among these factors, the body position (Krasnow & Bloomfield 1976), temperature (Surawicz 1995), blood electrolytes (Surawicz 1995), recording technique, lead choice (McLaughlin *et al*. 1996), the subject's age and gender (Stramba-Badiale *et al*. 1997) and finally the individual genetic profile are important and should be considered in any investigations (figure 1). Most of these factors can be controlled except for genetic predisposition, which is unknown in most cases if no prior congenital diseases are suspected. The regulating mechanisms of VR are apparent when assessing the VR dependence and adaptation to heart rate (HR; Franz *et al*. 1988). These dependences are controlled through the central nervous system (CNS; Magnano *et al*. 2002). These mechanisms represent confounding factors that are difficult to control because they are different across individuals and they are not yet thoroughly understood.

Figure 1 summarizes these factors and visually schematizes their known interactions. The right part of figure 1 describes the electrocardiographic expressions of the presence of an arrhythmogenic substrate or myocardial vulnerability to ventricular cardiac arrhythmias. The QT/QTc interval prolongation (Moss *et al*. 1985), abnormal repolarization heterogeneity (Restivo *et al*. 2004) and exercise-induced ST elevation/depression reveal the existence of an arrhythmogenic substrate (Antman *et al*. 2004), while T-wave alternans (Rosenbaum *et al*. 1994), abnormal QT/RR dynamicity, increased QT (Berger 2003) and T-wave variability (Couderc *et al*. 2007*b*) and ST changes are associated with the presence of myocardial vulnerability.

### (a) Measuring the ventricular repolarization from the body surface electrocardiogram

The electrocardiographic signals can be polluted by three independent sources of noise and artefacts: (i) muscular activities, electrode movement and respiration (baseline wander, i.e. the drift of the isoelectric line and amplitude modulations of the ECG signal), (ii) external electromagnetic signals of electrical equipment in close proximity, and (iii) interference from the power supply lines (50–60 Hz). Thus, a set of pre-processing steps is necessary to increase the signal-to-noise ratio. Several types of denoising techniques have been employed, and their definitions usually vary according to the type of analysis considered.

#### (i) ECG signal pre-processing

In general, the use of filters with nonlinear phase response is not acceptable because these filters drastically modify the ECG waveform (and can affect the ECG diagnosis) while filters with linear phase response (or zero phase response) are widely used. The general technical specifications of bandwidth and digital ECG processing (for clinical use) have been defined for more than a decade (Bailey *et al*. 1990), but the design of pre-processing ECG techniques remains an active field striving to improve the quality of current technologies. Indeed, in addition to classical digital filters (IIR and FIR), there is a plethora of published methods designed to filter the ECG signal and its VR, which include Wiener and wavelet filtering, principal component analysis, neural networks, Lyapunov exponents, entropy and model-based filtering, among others.

The baseline wander usually lies below 0.5 Hz but can spread to higher frequencies, leading to common frequencies with the T-wave signal (located below 60 Hz). The conventional adaptive algorithms represent an interesting alternative; they fit particularly well to the ECG pre-processing because they do not require a priori information about the relationship between noise and ECG signal (Widrow *et al*. 1975; Yunfeng *et al*. 2007; Tianjian *et al*. 2008). The main drawback of adaptive filter modelling techniques is their need to be adjusted when used on the ECGs of individuals with different cardiac states. In such cases, the wave delineation techniques and filter band construction are rarely applicable across populations.

#### (ii) ECG beat annotation

Analysing the VR interval requires a careful annotation of the cardiac beats. This step is crucial because the morphology of the repolarization segment on the surface ECG depends directly on the timing, pathway and regulation of the underlying repolarization activity. For instance, the T-wave of ventricular ectopic (or premature) beats has, in general, peculiar morphology because these beats are triggered inside the ventricles. Their repolarization process propagates along very different pathways than in normal sinus beats. A description of the techniques used to annotate cardiac beats is beyond the scope of this review but their description can be found in Bianchi *et al*. (2002).

#### (iii) ECG wave extraction and the QT interval

The identification of the different complexes and waves of the ECG signal is necessary when implementing an analysis of the VR. Fiducial points, such as QRS onset, R peak, J point and T-wave offset (figure 1), are required and need to be determined precisely. It is noteworthy that the end of the VR process does not correspond to the end of the T-wave in an ECG lead; rather, it reflects the projection of the ventricular repolarization front onto the associated lead axis. This emphasizes the role of lead selection in repolarization analyses.

Computerized methods for automatically measuring the QT interval are numerous. Willems (1986) compared QT interval measurements from 11 different algorithms and manual measurements: the standard deviation of differences between the QT measurements varied between 8 and 28 ms. The inter-observer difference was 15 ms. Savelieva *et al*. (1998) reported shorter QT interval measurements when based on an automatic method (least-squares fit technique) in comparison with manual measurements. In lead V1, automatic measurements were 25 ms shorter than manual measurements. The errors in the measurements of the automatic method are mainly associated with low-amplitude T-wave (Murray *et al*. 1994; Lund *et al*. 2002), abnormal morphology of T-wave (biphasic and notched T-wave) and the presence of a U-wave. The definition of the U-wave remains vague for most cardiologists, and where some would identify U-waves, others would see an abnormal T-wave morphology characterized by a notched shape rather than a QT–U complex. This might explain why computerized U-wave analyses are rather scarce today.

Interestingly, engineers have considered mimicking cardiologists' QT measurements using supervised techniques and probabilistic computation (Hughes & Tarassenko 2004; Andreao *et al*. 2006). Such concepts are interesting but they require access to large learning sets of QT measurements, which might be difficult. An international study evaluated the ability of physicians to identify an abnormal QT interval from ECG traces. Correct classification of all QT intervals as either ‘long’ or ‘normal’ was achieved by 96 per cent of QT experts and 62 per cent of arrhythmia experts, but by less than 25 per cent of cardiologists and non-cardiologists (Viskin *et al*. 2005). Consequently, the quality of QT interval measurements requires experience and a good understanding of the ECG signal.

### (b) Regulation of the ventricular cardiac repolarization

The risk of ventricular arrhythmias and sudden cardiac death in cardiac patients is further enhanced by changes in autonomic regulation of the heart. Observations in patients with heart failure suggested that an increased parasympathetic innervation is associated with poor prognosis (Van de Borne *et al*. 1997), while beta-adrenergic receptor blockers reduce the mortality of post-infarction patients (Shusterman *et al*. 1998).

The investigational work of Nollo *et al*. (1992) represents a pioneering effort to understand the dual control of the autonomic nervous system (ANS) on the cycle length and the VR. This investigation described a common synchronicity between the beat-to-beat variability of the VR interval (QT and RT intervals) and the heart-rate variability (RR intervals) based on the comparison of the location of the peaks of their respective power spectral density functions. Later, this result was confirmed by Speranza *et al*. (1993) and Lombardi *et al*. (1996). To reduce the repolarization variability due to measurements, Merri *et al*. (1989) investigated the variability of intervals inside the QT interval, focusing their analysis on RTm versus RR coupling and demonstrating that the QT dependence to HR primarily affects the early portion of the T-wave (RTm). Subsequently, Porta *et al*. (1998*b*) confirmed this observation and demonstrated that RTm was less robust than RTend to ECG amplitude modulation due to respiration, thus suggesting that a fraction of the RTm variability might be a consequence of the presence of respiratory amplitude modulation. Also, this study revealed an increased robustness of RTm measurements to broadband noise in comparison with RTend.

It is noteworthy that most of these investigations rely on ECGs from healthy individuals in which the interval between the apex and the end of the T-wave is known to be independent of HR.

#### (i) Cardiac repolarization and heart rate

The most obvious and not yet fully characterized physiological factor influencing the VR is the HR. In clinical studies, power-law models QTc(*i*)=*α*+*β*×RR(*i*−1)^*γ*^ are used despite the fact that the QT–RR relationship is described as a subject-dependent phenomenon (Couderc *et al*. 2000). Fridericia's (QTc=QT×RR^−1/3^) and Bazett's (QTc=QT×RR^−1/2^) are the most common examples of rate correction formulae, where RR is expressed in seconds and is the interval from the beats prior to the QT interval. The issue of individual specificity of the QT–RR relationship is not so relevant to general clinical cardiology but it becomes important when investigating the cardiac safety of new chemical compounds in which very small QTc prolongation is considered a warning sign of drug cardiotoxicity. Obviously, it becomes crucial when investigating the level of cardiotoxicity of drugs with effects on the autonomic regulation of the heart, such as antidepressant drugs. The QT interval duration is not the only aspect of the repolarization process affected by the HR. The amplitude of the T-wave is also significantly rate dependent. We investigated the T-wave amplitude and its rate in continuous 12-lead digital Holter recordings from 37 healthy individuals (Couderc *et al*. 2007*a*). The value of the slope characterizing the relationship between the amplitude of the T-wave and the RR intervals was 0.55±0.29 μV ms^−1^ in these individuals. This interplay between HR and the amplitude of the T-wave is very often carelessly neglected in analysis of the repolarization signal. Finally, the QT interval and repolarization morphology are assumed to be dependent uniquely on the previous single RR interval (or to be in steady state). The next section will describe how VR intervals are dependent on the history of HR, namely the previous sequence of RR intervals, the role of the ANS and how their relationship is being modelled.

#### (ii) QT interval adaptation to heart-rate changes and autonomic regulation

From clinical observations, findings around the role of the ANS on the QT interval are difficult to reconcile. Based on pharmacological autonomic blockade, it was found that sympathetic stimulations prolong the QT interval, and vagal stimulations shorten it (Extramiana *et al*. 2000). Opposite results were found by Browne *et al*. (1983) and Bellavere *et al*. (1988). Other authors compared QT intervals between diurnal and nocturnal periods at a similar HR (60 bpm). This difference was consistently close to 18 ms between studies when subtracting nocturnal from diurnal values. Thus, the predominance of the sympathetic tone within the vagosympathetic balance seems to be associated with an increased QT interval. When considering the analysis of the QT–RR slope between day and night, studies have shown that the slope is steeper during daytime. Our group reported slope values between day and night equal to 0.16±0.08 versus 0.12±0.12 (*p*=0.0001) based on one of the largest groups of 24 h Holter ECGs including 204 healthy subjects (Couderc *et al*. 2005).

Meanwhile, experimental observations have revealed a time of adaptation of the QT interval to HR. One of these experiments was based on measurements of endocardial AP duration in 17 subjects acquired under a specific pacing protocol designed by Franz *et al*. (1988). Their protocol included abrupt sustained rate acceleration and deceleration that evidenced the non-steady state and steady state of the VR. The time of steady-state adaptation was found to be several minutes (2–3 min). Repolarization restitution time was shown to be different between individuals (Pueyo *et al*. 2003), and the repolarization adaptation to be faster when the heart rhythm is increased than when it is decreased, leading to a ‘hysteresis’ effect (Lau *et al*. 1988). Analysis of the shape of such hysteresis has been evaluated as a marker of an increased risk of ventricular arrhythmias in patients exposed to potentially dangerous drugs (Fossa *et al*. 2007).

This QT interval dependence on the previous history of RR interval has been investigated by various groups striving to model this relationship in order to produce an improved rate correction technique (in comparison with power-law models) while better characterizing the role of ANS regulation on cardiac cells.

Porta *et al*. propose a dynamic bivariate linear parametric model to decompose the RT variability into RR- and non-RR-related variations with a model defined by

(2.1) $$\text{RT}(i)=A_{11}\text{RT}(i)+A_{12}\text{RR}(i)+n_{\text{RT}}(i).$$

The model encompasses two possible memory mechanisms: the history of RR and the previous RT period. *A*_11_ and *A*_12_ represent weighting factors based on *n* previous beats (all-zero polynomial of order *n*). The *n*_RT_(*i*) term is the noise affecting the RT interval independently from the RR variation. Based on a multivariate spectral decomposition, the resulting RT–RR transfer function shows a strong ‘fast dynamics component’ related to the immediately preceding RR including the low- (approx. 0.1 Hz) and high-frequency (approx. 0.2 Hz) concentrations, but more importantly ‘a very slow dynamic component’ unrelated to RR (Porta *et al*. 1998*a*).

Halamek *et al*. investigated the QT–RR coupling with a similar concept but using a lower-order model based on a recursive relation of the form

(2.2) $$\text{QT}(i)=b_{1}\text{RR}(i)+\cdots +b_{n}\text{RR}(i-n+1)-a_{1}\text{QT}(i-1)-\cdots -a_{m}\text{QT}(i-m)+e(i),$$

where *e*(*i*) is a random output. The order of such a model was defined by *K*=*n*+*m*. The optimal transfer function was based on three criteria, including a minimization of the mean level of standard deviation of a residual factor (equation (2.3)), a reduction of the mean level of the relative variability not described by the model (defined as Rerr=std(QT−QTm)/std(QT)), and finally a reduced variability of parameters in the model:

(2.3) $$r=\sqrt{\frac{\sum_{i=1}^{N}{\left(\text{QT}(i)-\text{QT}\text{m}(i)\right)}^{2}}{N-K}},$$

where *N* stands for the total number of analysed beats and QTm is the computed QT for beat *i*.

Based on ECGs including large heart-rate variations, Halamek's work led to a three-parameter model including: (i) gain for slow RR variability, (ii) gain for fast RR variability, and (iii) QT delay. This method performed much better than power-law correction formulae in ECGs with very unstable heart-rate state and when applied to patients with a pacemaker, in whom such a model could explain 70 per cent of the QT variability (Halamek *et al*. 2007).

Pueyo *et al*. presented a full beat-to-beat adaptation analysis using a method based on weighted average RR measurements with weight specifically defined for each cardiac beat (being a linear FIR time-variant filter, *h*(*i*)), coupled with a time-varying nonlinear function of the averaged preceding RR measurements. The global system is described as follows:

QT(i)=g(z(i),a(i))+v(i),

where *Z*(*i*)=*h*(*i*)^T^RR(*i*), and *v*(*i*) represents the added noise uncorrelated with the RR series. The function *g*(..,*a*(*i*)) is used to account for the different adaptation characteristics along the ECG recording. A recent review by Pueyo *et al*. (2009) on her technique was recently released in this journal.

The non-RR-related variation of the QT was consistently revealed by several authors. Almeida *et al*. analysed simulated data and 24 real segments of ECG signals using similar techniques to that reported by Porta *et al*. Segment-specific models of QT–RR coupling were developed. The study concluded on the presence of significant QT variability uncorrelated with sinus regulation, and suggesting the presence of direct regulation of the VR by the ANS (Almeida *et al*. 2006). Lombardi *et al*. (1998) using the model from Porta *et al*. reported an RT variability fraction driven by HRV significantly greater in young subjects than in post-myocardial infarction patients and age-matched control subjects. The rate dependence of VR remains to be fully elucidated, but one recognizes the role of intrinsic characteristics of myocardial fibres and/or modulation by the ANS (Coumel & Maison-Blanche 2003). The QT–RR coupling is of great interest and I would emphasize the interest from the medical community in the uncorrelated part of the beat-to-beat variability of the QT interval reflecting a possible impairment of the VR when it is abnormally increased. This assumption is strengthened following the growing number of studies investigating repolarization variability for the risk stratification of cardiac patients with a variety of clinical conditions. Various methods were designed to investigate the role of VR instability as a surrogate marker of cardiac vulnerability: QT variability (Berger 2003), T-wave alternans (Rosenbaum 2008) and T-wave morphology instability (Couderc *et al*. 2007*b*).

## 3. Quantifications of the morphology of the ventricular repolarization segment and their clinical applications

Instead of controlling the effect of the various factors known to affect the QT interval and the amplitude of the T-wave, investigators have considered alternatives through the development of novel repolarization quantifiers less dependent on HR. Clinically, it is well accepted that a QTc interval beyond 500 ms is a clear predisposing factor for the occurrence of dangerous ventricular arrhythmias such as the *torsades de pointes*, but the presence of a QT interval duration below 500 ms is associated with a less clear-cut risk. The development of new biomarkers complementing the QT interval measurements for the identification of a predisposition to ventricular arrhythmias in congenital LQTS and for the assessment of drug cardiotoxicity is of major interest. The objective of this section is to review a set of methods designed to quantify the morphology of the repolarization interval.

### (a) Techniques for the quantification of T-wave morphology

Early methods for the quantification of the repolarization interval focused on the T- and U-wave shapes from the scalar leads. The method from Padrini *et al*. (1995) defined the ECG signal (*S*) generated by a set of cells (*C*) as

(3.1) $$S(t)=\sum_{c\in C}\xi_{c}{\text{AP}}_{c}(\delta_{c}+t),$$

where AP_*c*_ is the AP of the cell *c*; *δ*_*c*_ is the time of excitation; and *ξ*_*c*_ denotes a correcting factor for weighting the effect of distance between the electrodes and the cardiac cell, and the surrounding tissue resistance. In the working assumptions of their model, the differences in excitation times *δ*_*c*_ and *ξ*_*c*_ terms were considered to be reflected in the modelled phases 2 and 3 of the cell AP. A Hill's shape was used in this specific work to generate the decomposition curve. Hill's equation is described as

(3.2) $$A(t)=A(\frac{t^{n}}{T_{50}^{n}+t^{n}}).$$

The best-fitting procedure was carried out based on the Levenberg–Marquardt algorithm (Marquardt 1963). They modelled the T–U complexes using

(3.3) TU(t)=[S1(t)−S2(t)]+[L1(t)−L2(t)],

where S1, S2, L1 and L2 are sigmoidal equations representing the plateau and down stroke of a cardiac AP. Thus, the left components (S1 and S2) represent the T-wave and the right components (L1 and L2) correspond to the contribution to the U-wave. The T-wave was characterized based on the following four parameters:

(3.4) $$A_{\text{max}}=A_{\text{inf}}\frac{t_{\text{max}}^{n}}{\left(T_{50}^{n}+t_{\text{max}}^{n}\right)},D_{95}=t_{\text{max}}-{\left[\frac{0.05A_{\text{max}}T_{50}^{n}}{A_{\text{inf}}-0.05A_{\text{max}}}\right]}^{1/n},D_{5}=t_{\text{max}}-{\left[\frac{0.95A_{\text{max}}T_{50}^{n}}{A_{\text{inf}}-0.95A_{\text{max}}}\right]}^{1/n},A-\text{ratio}=\frac{A_{\text{max}}(\text{L}1)+A_{\text{max}}(\text{L}2)}{A_{\text{max}}(\text{S}1)+A_{\text{max}}(\text{S}2)}.$$

The *A*-ratio quantified the relative contribution of the U- and T-wave components. These two parameters, in addition to Hill's coefficient, were used to characterize the morphology of the ECGs in two independent studies. A first study investigated the ability of the technique to appropriately fit real ECG signals (T–U complexes) in healthy individuals (*n*=22) and in cardiac patients (*n*=12) including individuals with the LQTS and/or hypertrophic cardiomyopathy (Padrini *et al*. 2001). The indices of similarity were based on the absolute error between modelled signal (approx. 5 μV among all leads) and the correlation between the signals and its modelled version (superior to 0.99).

Another more recent algorithm was designed by Dubois *et al*. (2007). Its application to the characterization of the T-wave morphology was pursued by Badilini *et al*. (2008). This modelling is based on the Gaussian mesa function (GMF) defined by

(3.5) $$\begin{array}{c} B(X,\mu ,\sigma_{1},\sigma_{2},A)=A\;\text{exp}\left(-0.5{\left(\frac{x-\left(\mu -\sigma_{\text{L}}/2\right)}{\sigma_{1}}\right)}^{2}\right)\;\text{if}\;x\leq \mu -\sigma_{\text{L}}/2,\\ B(X,\mu ,\sigma_{1},\sigma_{2},A)=A\;\text{if}\;\mu -\sigma_{\text{L}}/2\leq x\leq \mu +\sigma_{\text{L}}/2,\\ B(X,\mu ,\sigma_{1},\sigma_{2},A)=A\;\text{exp}\left(-0.5{\left(\frac{x-\left(\mu -\sigma_{\text{L}}/2\right)}{\sigma_{2}}\right)}^{2}\right)\;\text{if}\;x\geq \mu +\sigma_{\text{L}}/2, \end{array}$$

where *A* represents the amplitude of the plateau of the function; *μ* is the time location for the function; *σ*_L_ is the length of the plateau phase; and *σ*_1_ and *σ*_2_ denote the standard deviation of the ascending and descending Gaussian functions, respectively, forming the overall shape of the mesa function. The fitting algorithm to the ECG signal was based on generalized orthogonal forward regression (Broyden 1970). This method is used for modelling all the waves and complexes of the cardiac beats, leading to a set of six GMF models.

A third technique was reported by Kanters *et al*. (2004) and was based on Hill's equation, similar to the work described by Padrini *et al*. described above. However, this group used a simplified approach and reported the coefficients of Hill's formula.

**Figure 2:**
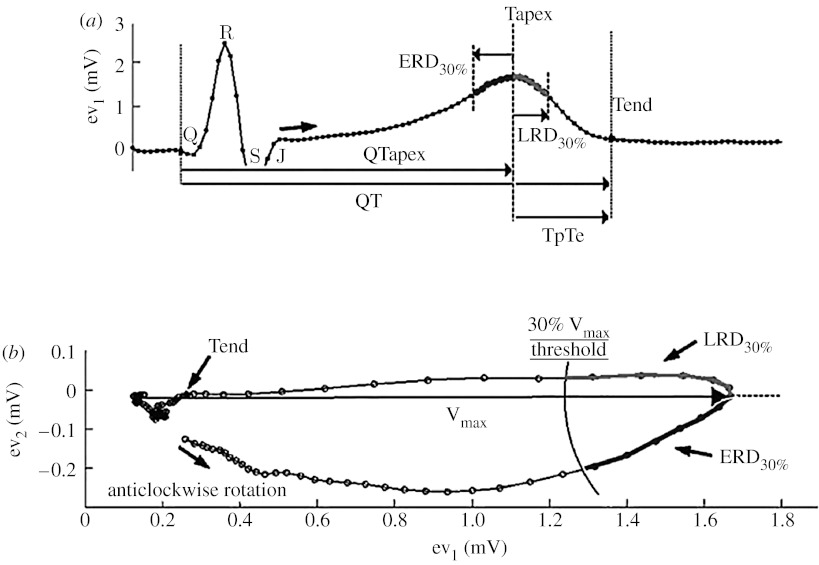
(*a*,*b*) Description of the vectocardiographic measurements of the early (ERD) and late (LRD) repolarization measurements (see text for detailed description).

Other pioneering works related to T-wave morphology measurements did not rely on modelling techniques but rather quantified the distribution of time and amplitude across the T-wave. Merri *et al.* designed such a method, which was subsequently applied to both LQTS patients, healthy individuals (Merri *et al*. 1989) and drug-induced LQTS (Couderc *et al*. 2003). This method relied on the measurement of the cumulated area under the T-wave from scalar ECGs. Subsequently, the techniques relied on ‘global’ leads computed from all available non-redundant leads (eight leads: I, II, V1–V6 or orthogonal leads X, Y and Z) to reduce the effect of physical movement and the variability associated with lead placements. The global or eigen leads were based on the computation of the singular value decomposition and principal component analysis. The repolarization signal represented in the space defined by the three first components (ev_1_, ev_2_ and ev_3_) is a loop (rather than a cloud because of the intrinsic organization of the repolarization process). Interestingly, most of the repolarization energy is inscribed into a single plane (ev_1_└ev_2_), also called the preferential plane of the T-loop (figure 2*b*). A normal loop in its preferential plane is narrow (*λ*_2_/*λ*_1_<1) and flat; a more rounded T-loop and increased planarity are associated with increased repolarization heterogeneity (Priori *et al*. 1994; Zareba *et al*. 2000).

Our group proposed a set of new repolarization measurements independent of the localization of the end of the T-wave, and measuring changes in the morphology of the T-loop. In this method, the arbitrarily chosen reference point was the time when the heart vector reaches its maximum value (MV). MV is detected at time *t*=*T*_MV_, at which

(3.6) $$\text{MV}=\text{max}\langle \sqrt{{\left\{{\text{ev}}_{1}(t)-{\text{ev}}_{1}(T_{\text{Q}})\right\}}^{2}+{\left\{{\text{ev}}_{2}(t)-{\text{ev}}_{2}(T_{\text{Q}})\right\}}^{2}}\rangle$$

is fulfilled, where $\text{VECG}(t)={\text{ev}}_{1}(t)\vec{i}+{\text{ev}}_{2}(t)\vec{j}$ and *T*_Q_ is the time coinciding with the beginning of the QRS complex.

As shown in figure 2, the so-called early and late repolarization duration (ERD_30%_, LRD_30%_) intervals are centred on the time of MV. Precisely, ERD=*T*_MV_−*T*_E_, where *T*_E_ is the value for *t* at which equation (3.7) is fulfilled,

(3.7) $$\|\text{VECG}(t)-\text{VECG}(T_{\text{MV}})\|=\text{MV}\times 30\%,$$

and LRD=*T*_L_−*T*_MV_, where *T*_L_ is the value for *t* at which equation (3.8) is fulfilled,

(3.8) $$\|\text{VECG}(T_{\text{MV}})-\text{VECG}(t)\|=\text{MV}\times 30\%.$$

The early repolarization duration (ERD_30%_) and the late repolarization duration (LRD_30%_) measure the time needed for the heart vector to vary from its maximum length to a time point corresponding to a 30 per cent reduction of its maximum length during the repolarization process. LRD_%_ is a measure towards the next cardiac beat and ERD_%_ is directed towards the J point of the same cardiac beat (figure 2). The duration of these time intervals increases when the heart vector slows down and/or the roundness of the T-loop increases. Consequently, these parameters measure the repolarization duration (reflected in the velocity of the heart vector) and repolarization heterogeneity (reflected in the path of the heart vector or T-loop morphology).

### (b) Clinical significance of abnormal repolarization morphologies

Kanters and Struijk investigated the role of T-wave morphology in ECGs from patients with different types of LQTS mutations (associated with gene-specific dysfunctions of cardiac ion kinetics), assuming that different ion dysfunctions would lead to different morphologies, as experimentally documented by Moss *et al*. (Moss *et al*. 1995; Struijk *et al*. 2006). These authors showed that, among the three investigated leads II, V2 and V5, lead V2 showed the most differences between the groups. They reported observations corresponding to smaller T-wave amplitude in LQT2 than in LQT1 patients and higher amplitude in LQT1 than in healthy individuals (Struijk *et al*. 2005). No heart-rate dependence of Hill coefficients was found. Vaglio *et al*. (2008) have studied the morphology of the T-loop and their results suggested that the roundness of the T-wave is significantly different (*p*<0.05) between LQT1, LQT2 and healthy individuals (healthy, 0.15±0.06; LQT1, 0.22±0.10; and LQT2, 0.36±0.17; *p*<0.05). Badilini *et al*. (2008) implemented similar investigations in another independent dataset of LQTS patients (50 LQT1, 50 LQT2 and 38 controls) showing gene-specific repolarization morphologies. The T-wave amplitude was significantly smaller in LQTS patients versus the controls and between the two types of LQTS (LQT1, 974±501; LQT2, 749±384; control, 1043±451 μV; *p*<0.05.

In the arena of drug safety, more subtle changes in the repolarization process have to be detected than those observed in most patients with congenital LQTS. Several authors have investigated independently the repolarization morphology in ECGs from 38 healthy individuals exposed to a drug called sotalol. These ECGs were recorded during a study evaluating the agreement between various QT interval techniques (Sarapa *et al*. 2004). Sotalol blocks the specific repolarizing ion currents involved in the termination of the AP of the heart cells (leading to a prolongation of the QT interval and an increased ventricular heterogeneity). This drug is associated with dangerous ventricular arrhythmias. Regardless of the method employed to quantify the VR, they all revealed that sotalol prolonged the QTc interval duration (approx. 50 ms) and profoundly changed the morphology of the T-wave. To complement these results, we implemented a morphological analysis of repolarization in healthy individuals exposed to moxifloxacin (Couderc *et al*. 2008). Moxifloxacin is a drug that blocks the same repolarizing current as sotalol but in a milder way. This drug is considered safe at normal dose. Based on ECGs from 40 healthy individuals exposed to moxifloxacin, significant changes in the early part of the T-wave, but not the final part, were observed. This result emphasizes that drug-induced QT prolongation might be associated with different types of prolongation of the repolarization signals. Investigating the way the delay of the QT interval occurs within the repolarization interval might be helpful for dissociating dangerous from harmful drugs for the repolarization process of the heart.

These studies revealed the ability of digital methods to quantify the morphology of the VR signal. They highlight the complementary information between the QT interval and the repolarization morphology. In addition, most of these parameters are reported with a negligible or weak dependence to HR (Andersen *et al*. 2008; Couderc *et al*. 2008) but their dependence on the autonomic regulation remains to be elucidated. Finally, the link between cardiac arrhythmias and the presence of abnormal morphologies should be demonstrated; the next section will discuss this point.

### (c) Cardiac events, abnormal morphology and gender

Women tend to have a longer QTc interval than men, and women tend to have a stronger reaction to a drug prolonging the QTc interval (and more adverse events). Lehmann & Yang (2001) investigated the differences in T-wave morphology between genders; they reported a ‘sexual dimorphism’ described as a reduced repolarization velocity of the ascending part of the T-wave in females. In LQT2 patients and their relatives, the same initial part of the T-wave was shown to be useful for discriminating patients with and without mutation but normal QT interval duration (Couderc *et al*. 2006). The interval prior to the apex of the T-wave was used to identify healthy subjects exposed to moxifloxacin, a drug that can generate arrhythmias at very high concentration. In two independent investigations involving a group of patients with and without a history of drug-induced arrhythmias (Kaab *et al*. 2003; Couderc *et al*. 2009), the results suggested that sotalol-induced QTc prolongation was significantly more pronounced in the group of individuals with a history of drug-induced arrhythmias (*p*<0.06) than in patients without such a history. More interestingly, sotalol significantly prolongs the terminal part of the repolarization interval in patients with a history of arrhythmias: TpTe interval (36±41 versus 6±20 ms, *p*=0.01) and LRD_70%_ (27±30 versus 8±13 ms, *p*=0.03). These observations support the hypothesis that different changes in morphology might be associated with different clinical outcomes.

## 4. Conclusion

Over the past 60 years, the era of digital electrocardiography has spawned a large number of methods revealing electrophysiological mechanisms that one could not have accessed without the help of computerized technologies. Long-term ECG recordings and digital signal processing technologies unveiled the regulation of the VR by the central nervous system. Later its impairment was found to be associated with lethal risk. This led to a set of techniques to measure the rate-independent variability of the VR and to evaluate its value as a marker of arrhythmogenic risks. In parallel, the assessment of an abnormal VR process based on QT interval duration measurements became unsatisfactory. In such a context, the extension of the analysis of the VR signal to information inside the QT interval (morphology) was proposed as an alternative. How to best measure these changes remains to be decided, and how these morphology changes should be translated in cardiac risks is an exciting challenge.

As a final remark, one would emphasize that the development of these technologies will depend on the accessibility of relevant ECG data. Even if their design may not require a large set of ECG recordings, their validation does. Access to such an amount of data might be challenging, so I believe that initiatives such as the PhysioBank (www.physionet.org) and the Telemetric and Holter ECG Warehouse (www.thew-project.org) are valuable resources for researchers and engineers.

## References

[bib1] Almeida R., Gouveia S., Rocha A.P., Pueyo E., Martinez J.P., Laguna P. (2006). QT variability and HRV interactions in ECG: quantification and reliability. IEEE Trans. Biomed. Eng.

[bib2] Andersen M., Xue J., Graff C., Kanters J., Toft E., Struijk J. (2008). New descriptors of T-wave morphology are independent of heart rate. J. Electrocardiol.

[bib3] Andreao R.V., Dorizzi B., Boudy J. (2006). ECG signal analysis through hidden Markov models. IEEE Trans. Biomed. Eng.

[bib4] Antman E.M. (2004). ACC/AHA guidelines for the management of patients with ST-elevation myocardial infarction—Executive summary: A report of the American College of Cardiology/American Heart Association Task Force on Practice Guidelines (Writing Committee to Revise the 1999 Guidelines for the Management of Patients With Acute Myocardial Infarction). Can. J. Cardiol.

[bib5] Badilini F., Vaglio M., Dubois R., Roussel P., Sarapa N., Denjoy I., Extramiana F., Maison-Blanche P. (2008). Automatic analysis of cardiac repolarization morphology using Gaussian mesa function modeling. J. Electrocardiol.

[bib6] Bailey J.J., Berson A.S., Garson A., Horan L.G., Macfarlane P.W., Mortara D.W., Zywietz C. (1990). Recommendations for standardization and specifications in automated electrocardiography: bandwidth and digital signal processing. A report for health professionals by an ad hoc writing group of the Committee on Electrocardiography and Cardiac Electrophysiology of the Council on Clinical Cardiology, American Heart Association. Circulation.

[bib7] Bellavere F., Ferri M., Guarini L., Bax G., Piccoli A., Cardone C., Fedele D. (1988). Prolonged QT period in diabetic autonomic neuropathy: a possible role in sudden cardiac death?. Br. Heart J.

[bib8] Berger R.D. (2003). QT variability. J. Electrocardiol.

[bib9] Bianchi A.M., Mainardi L.T., Cerutti S., Moss A.J., Stern S. (2002). Signal processing. Noninvasive electrocardiology: clinical aspects of Holter monitoring.

[bib10] Browne K.F., Prystowsky E., Heger J.J., Zipes D.P. (1983). Modulation of the Q–T interval by the autonomic nervous system. Pacing Clin. Electrophysiol.

[bib11] Broyden C. (1970). The convergence double rank minimization algorithm 2. The new algorithm. J. Inst. Math. Appl.

[bib12] Couderc J.P., Chevalier P., Fayn J., Rubel P., Touboul P. (2000). Identification of post-myocardial infarction patients prone to ventricular tachycardia using time–frequency analysis of QRS and ST segments. Europace.

[bib19] Couderc J.P., Zareba W., Moss A.J., Sarapa N., Morganroth J., Darpo B. (2003). Identification of sotalol-induced changes in repolarization with T wave area-based repolarization duration parameters. J. Electrocardiol.

[bib17] Couderc J.P., Xiaojuan X., Zareba W., Moss A.J. (2005). Assessment of the stability of the individual-based correction of QT interval for heart rate. Ann. Noninvasive. Electrocardiol.

[bib15] Couderc J.P., McNitt S., Xia J., Zareba W., Moss A.J. (2006). Repolarization morphology in adult LQT2 carriers with borderline prolonged QTc interval. Heart Rhythm.

[bib16] Couderc J.P., Vaglio M., Xia X., McNitt S., Wicker P., Sarapa N., Moss A.J., Zareba W. (2007a). Impaired T-amplitude adaptation to heart rate characterizes I(Kr) inhibition in the congenital and acquired forms of the long QT syndrome. J. Cardiovasc. Electrophysiol.

[bib18] Couderc J.P., Zareba W., McNitt S., Maison-Blanche P., Moss A.J. (2007b). Repolarization variability in the risk stratification of MADIT II patients. Europace.

[bib14] Couderc J.P., McNitt S., Hyrien O., Vaglio M., Xia X., Polonsky S., Moss A.J., Zareba W. (2008). Improving the detection of subtle i(kr)-inhibition: assessing electrocardiographic abnormalities of repolarization induced by moxifloxacin. Drug Saf.

[bib13] Couderc J.P., Kaab S., Hinterseer M., McNitt S., Xia X., Fossa A., Beckmann B.M., Polonsky S., Zareba W. (2009). Baseline values and sotalol-induced changes of ventricular repolarization duration, heterogeneity, and instability in patients with a history of drug-induced torsades de pointes. J. Clin. Pharmacol.

[bib20] Coumel P., Maison-Blanche P. (2003). Neuro-mediated repolarization abnormalities. Cardiac repolarization: bridging basic and clinical science.

[bib21] Dubois R., Maison-Blanche P., Quenet B., Dreyfus G. (2007). Automatic ECG wave extraction in long-term recordings using Gaussian mesa function models and nonlinear probability estimators. Comput. Methods Programs Biomed.

[bib22] Extramiana F., Tavernier R., Maison-Blanche P., Neyroud N., Jordaens L., Leenhardt A., Coumel P. (2000). Ventricular repolarization and Holter monitoring. Effect of sympathetic blockage on the QT/RR ratio. Arch. Mal. Coeur. Vaiss.

[bib23] Fossa A.A. (2007). Analyses of dynamic beat-to-beat QT–TQ interval (ECG restitution) changes in humans under normal sinus rhythm and prior to an event of torsades de pointes during QT prolongation caused by sotalol. Ann. Noninvasive. Electrocardiol.

[bib24] Franz M.R., Swerdlow C.D., Liem L.B., Schaefer J. (1988). Cycle length dependence of human action potential duration *in vivo*. Effects of single extrastimuli, sudden sustained rate acceleration and deceleration, and different steady-state frequencies. J. Clin. Invest.

[bib25] Halamek, J., Jurak, P., Villa, M., Novak, M., Vondra, V., Soucek, M., Frafia, P., Somers, V. K. & Kara, T. 2007 Dynamic QT/RR coupling in patients with pacemakers. In *EMBS 2007. Twenty-ninth Annu. Int. Conf. of the IEEE. Engineering in Medicine and Biology Society, Lyon, France, 2007*. 10.1109/IEMBS.2007.4352441 18002107

[bib26] Hughes, N. P. & Tarassenko, L. 2004 Automated QT interval analysis with confidence measures. In *Computers in Cardiology 2004, Chicago, IL*, vol. 31, pp. 765–768.

[bib27] Kaab S., Hinterseer M., Nabauer M., Steinbeck G. (2003). Sotalol testing unmasks altered repolarization in patients with suspected acquired long-QT-syndrome—a case-control pilot study using i.v. sotalol. Eur. Heart J.

[bib28] Kanters J., Fanoe S., Larsen L., Bloch Thomsen P., Toft E., Christiansen M. (2004). T wave morphology analysis distinguishes between KvLQT1 and HERG mutations in long QT syndrome. Heart Rhythm.

[bib29] Krasnow A.Z., Bloomfield D.K. (1976). Artifacts in portable electrocardiographic monitoring. Am. Heart J.

[bib30] Lau C.P., Freedman A.R., Fleming S., Malik M., Camm A.J., Ward D.E. (1988). Hysteresis of the ventricular paced QT interval in response to abrupt changes in pacing rate. Cardiovasc. Res.

[bib31] Lehmann M.H., Yang H. (2001). Sexual dimorphism in the electrocardiographic dynamics of human ventricular repolarization: characterization in true time domain. Circulation.

[bib33] Lombardi F., Sandrone G., Porta A., Torzillo D., Terranova G., Baselli G., Cerutti S., Malliani A. (1996). Spectral analysis of short term R–Tapex interval variability during sinus rhythm and fixed atrial rate. Eur. Heart J.

[bib32] Lombardi F., Colombo A., Porta A., Baselli G., Cerutti S., Fiorentini C. (1998). Assessment of the coupling between RTapex and RR interval as an index of temporal dispersion of ventricular repolarization. Pacing Clin. Electrophysiol.

[bib34] Lund K., Nygaard H., Kirstein P.A. (2002). Weighing the QT intervals with the slope or the amplitude of the T wave. Ann. Noninvasive. Electrocardiol.

[bib35] Magnano A.R., Holleran S., Ramakrishnan R., Reiffel J.A., Bloomfield D.M. (2002). Autonomic nervous system influences on QT interval in normal subjects. J. Am. Coll. Cardiol.

[bib36] Marquardt D. (1963). An algorithm for least-squares estimation of nonlinear parameters. SIAM J. Appl. Math.

[bib37] McLaughlin N.B., Campbell R.W., Murray A. (1996). Accuracy of four automatic QT measurement techniques in cardiac patients and healthy subjects. Heart.

[bib38] Merri M., Benhorin J., Alberti M., Locati E., Moss A.J. (1989). Electrocardiographic quantitation of ventricular repolarization. Circulation.

[bib39] Moss A.J., Schwartz P.J., Crampton R.S., Locati E., Carleen E. (1985). The long QT syndrome: a prospective international study. Circulation.

[bib40] Moss A.J. (1995). ECG T-wave patterns in genetically distinct forms of the hereditary long QT syndrome. Circulation.

[bib41] Murray A., McLaughlin N.B., Bourke J.P., Doig J.C., Furniss S.S., Campbell R.W. (1994). Errors in manual measurement of QT intervals. Br. Heart J.

[bib42] Nabauer M., Kaab S. (1998). Potassium channel down-regulation in heart failure. Cardiovasc. Res.

[bib43] Nollo G., Speranza G., Grasso R., Bonamini R., Mangiardi L., Antolini R. (1992). Spontaneous beat-to-beat variability of the ventricular repolarization duration. J. Electrocardiol.

[bib44] Padrini R., Butrous G., Camm A.J., Malik M. (1995). Algebraic decomposition of the TU wave morphology patterns. Pacing Clin. Electrophysiol.

[bib45] Padrini R., Butrous G., Statters D., Camm A.L., Malik M. (2001). Morphological algebraic models of the TU-wave patterns in idiopathic long QT syndrome. Int. J. Cardiol.

[bib46] Porta A., Baselli G., Caiani E., Malliani A., Lombardi F., Cerutti S. (1998a). Quantifying electrocardiogram RT–RR variability interactions. Med. Biol. Eng. Comput.

[bib47] Porta A., Baselli G., Lombardi F., Cerutti S., Antolini R., Del Greco M., Ravelli F., Nollo G. (1998b). Performance assessment of standard algorithms for dynamic R–T interval measurement: comparison between R–Tapex and R–T(end) approach. Med. Biol. Eng. Comput.

[bib48] Priori S.G., Napolitano C., Diehl L., Schwartz P.J. (1994). Dispersion of the QT interval. A marker of therapeutic efficacy in the idiopathic long QT syndrome. Circulation.

[bib50] Pueyo E., Smetana P., Laguna P., Malik M. (2003). Estimation of the QT/RR hysteresis lag. J. Electrocardiol.

[bib49] Pueyo E., Martinez J.P., Laguna P. (2009). Cardiac repolarization analysis using the surface electrocardiogram. Phil. Trans. R. Soc. A.

[bib51] Restivo M.R., Caref E.B., Kozhevnikov D., El-Sherif N. (2004). Spatial dispersion of repolarization is a key factor in the arrhythmogenicity of long QT syndrome. J. Cardiovasc. Electrophysiol.

[bib52] Rosenbaum D.S. (2008). T-wave alternans in the sudden cardiac death in heart failure trial population: signal or noise?. Circulation.

[bib53] Rosenbaum D.S., Jackson L.E., Smith J.M., Garan H., Ruskin J.N., Cohen R.J. (1994). Electrical alternans and vulnerability to ventricular arrhythmias. N. Engl. J. Med.

[bib54] Sarapa N. (2004). Electrocardiographic identification of drug-induced QT prolongation: assessment by different recording and measurement methods. Ann. Noninvasive. Electrocardiol.

[bib55] Savelieva I., Yi G., Guo X., Hnatkova K., Malik M. (1998). Agreement and reproducibility of automatic versus manual measurement of QT interval and QT dispersion. Am. J. Cardiol.

[bib56] Shusterman V. (1998). Autonomic nervous system activity and the spontaneous initiation of ventricular tachycardia. ESVEM investigators. Electrophysiologic study versus electrocardiographic monitoring trial. J. Am. Coll. Cardiol.

[bib57] Speranza G., Nollo G., Ravelli F., Antolini R. (1993). Beat-to-beat measurement and analysis of the R–T interval in 24 h ECG Holter recordings. Med. Biol. Eng. Comput.

[bib58] Stramba-Badiale M., Locati E.H., Martinelli A., Courville J., Schwartz P.J. (1997). Gender and the relationship between ventricular repolarization and cardiac cycle length during 24-h Holter recordings. Eur. Heart J.

[bib60] Struijk, J. J., Kanters, J. K., Andersen, M. P., Hardahl, T., Graff, C., Christiansen, M. & Toft, E. 2005 Classification of the long-QT syndrome based on discriminant analysis of the T-wave morphology. In *Computers in Cardiology 2005, Lyon, France*, vol. 32, pp. 511–514. 10.1007/s11517-006-0061-1 16937190

[bib59] Struijk J.J., Kanters J.K., Andersen M.P., Hardahl T., Graff C., Christiansen M., Toft E. (2006). Classification of the long-QT syndrome based on discriminant analysis of T-wave morphology. Med. Biol. Eng. Comput.

[bib61] Surawicz B. (1995). Electrophysiologic basis of ECGs and cardiac arrhythmias.

[bib62] Tianjian, C., Yong, Z., Chunyu, Y. & Ling, S. 2008 A new technique for ECG denoising using adaptive wavelet shrinkage. In *Computing, communication, control, and management, 2008. CCCM '08. ISECS international colloquium*, pp. 256–259.

[bib63] Vaglio M., Couderc J., McNitt S., Xia X., Moss A., Zareba W. (2008). A quantitative assessment of T-wave morphology in LQT1, LQT2, and healthy individuals based on Holter recording technology. Heart Rhythm.

[bib64] Van de Borne P., Montano N., Pagani M., Oren R., Somers V.K. (1997). Absence of low-frequency variability of sympathetic nerve activity in severe heart failure. Circulation.

[bib65] Viskin S. (2005). Inaccurate electrocardiographic interpretation of long QT: the majority of physicians cannot recognize a long QT when they see one. Heart Rhythm.

[bib66] Widrow B., Glover J.R., McCool J.M., Kaunitz J., Williams C.S., Hearn R.H., Zeidler J.R., Dong E., Goodlin R.C. (1975). Adaptive noise cancelling: principles and applications. Proc. IEEE.

[bib67] Willems, J. L. 1986 Common standards for quantitative electrocardiography. CSE 6th Progress Report. CSE 86-12-08, 139. Leuven, ACCO.

[bib68] Wu, Y., Rangayyan, R. M. & Ng, S.-C. 2007 Cancellation of artifacts in ECG signals using a normalized adaptive neural filter. In *EMBS 2007. Twenty-ninth Annu. Int. Conf. of the IEEE. Engineering in Medicine and Biology Society, Lyon, France*, pp. 2552–2555. 10.1109/IEMBS.2007.4352849 18002515

[bib69] Zareba, W., Couderc, J. P. & Moss, A. J. 2000 Automatic detection of spatial and temporal heterogeneity of repolarization. In *Dispersion of ventricular repolarization: state of the art* (eds S. Olsson, S. Yuan & J. Amlie), pp. 85–108. Armonk, NY: Futura.

